# The Small Molecule Inhibitor of the Type III Secretion System Fluorothiazinone Affects Flagellum Surface Presentation and Restricts Motility in Gram-Negative Bacteria

**DOI:** 10.3390/antibiotics14080820

**Published:** 2025-08-11

**Authors:** Alexey Slonov, Mariam Abdulkadieva, Egor Kalinin, Natalya Bondareva, Lydia Kapotina, Svetlana Andreevskaya, Natalia Shevlyagina, Anna Sheremet, Elena Sysolyatina, Vladimir Zhukhovitsky, Mikhail Vasiliev, Oleg Petrov, Svetlana Ermolaeva, Nailya Zigangirova, Alexander Gintsburg

**Affiliations:** 1 Department of Infections with Natural Foci, Gamaleya National Research Center of Epdemiology and Microbiology, Gamaleya st. 18, Moscow 123098, Russia; alex.d.slonov@gmail.com (A.S.); maryam094@yandex.ru (M.A.); kalinin.egor@bk.ru (E.K.); demiurg_84@mail.ru (E.S.); 2 Department of Dusty Plasmas, Joint Institute for High Temperatures of the Russian Academy of Sciences, Moscow 125412, Russia; mixxy@mail.ru (M.V.); ofpetrov@ihed.ras.ru (O.P.); 3 Department of Medical Microbiology, Gamaleya National Research Center of Epdemiology and Microbiology, Gamaleya st. 18, Moscow 123098, Russia; nataliia.d@mail.ru (N.B.); lidiya.kapotina57@mail.ru (L.K.); anna-pimenova@mail.ru (A.S.); zigangirova@mail.ru (N.Z.); 4 Department of Bacterial Infections, Gamaleya National Research Center of Epdemiology and Microbiology, Gamaleya st. 18, Moscow 123098, Russia; hacaranda@yandex.ru (S.A.); nataly-123@list.ru (N.S.); zhukhovitsky@rambler.ru (V.Z.); 5 Department of Clinical Laboratory Diagnostics, Russian Medical Academy of Continuous Professional Education (RMANPO), Moscow 125993, Russia; 6 Department of Bacterial Genetics and Molecular Biology, Gamaleya National Research Center of Epdemiology and Microbiology, Gamaleya st. 18, Moscow 123098, Russia; gintsburg@gamaleya.ru; 7 Department of Infectious Diseases and Virology, Institute of Professional Education, First Moscow State Medical University Named After I. M. Sechenov, Gamaleya st. 18, Moscow 123098, Russia

**Keywords:** Fluorothiazinone, flagellum assembly, bacterial motility, type III secretion system, Gram-negative bacteria, anti-virulence agent

## Abstract

Background/Objectives: Fluorothiazinone (FT), a small molecule of the 2,4-disubstituted-4H-[1,3,4]-thiadiazine-5-one class, is known to inhibit the type III secretion system (T3SS) in Gram-negative bacteria and has shown therapeutic potential in animal models and clinical trials. Given the evolutionary relationship between the T3SS and the bacterial flagellar apparatus, this study aimed to investigate the effects of FT on bacterial motility and flagellum assembly. Methods: Motility was assessed in *Pseudomonas aeruginosa*, *Proteus mirabilis*, pathogenic *Escherichia coli*, and *Listeria monocytogenes* using a semisolid agar assay and a microfluidic motility system. The mechanism of FT’s action was further examined through time-course analysis, Western blotting of surface flagella proteins, and transmission electron microscopy (TEM). Results: FT inhibited motility of *P. aeruginosa*, *P. mirabilis*, and *E. coli* in a dose-dependent manner, while *L. monocytogenes* motility remained unaffected. The inhibitory effect was not immediate but delayed 2–3 h post FT addition. Western blotting revealed the absence of surface flagella in EHEC grown with FT, and TEM confirmed structural disruption of flagella in *P. mirabilis*. Conclusions: FT selectively inhibits flagellum-based motility in Gram-negative bacteria. Obtained data suggested FT interference with flagellum biosynthesis rather than disruption of rotation. Motility inhibition can contribute to FT therapeutic effects on Gram-negative bacterial infections.

## 1. Introduction

Flagellar motility is an important feature of many pathogenic bacteria, including *Escherichia coli*, *Salmonella typhimurium*, *Pseudomonas aeruginosa*, *Vibrio cholerae*, *Proteus mirabilis*, *Helicobacter pylori*, *Listeria monocytogenes*, and some others [1,2,3,4]. Motility is critical for pathogen survival in the external environment and is often associated with virulence. Flagellum-mediated motility has been shown to contribute to the ascending dissemination and colonization of the upper urinary tract by uropathogenic *E. coli* (UPEC) and *P. mirabilis* [5,6]. Monotrichous polar flagella are essential for biofilm formation, colonization, and virulence in *V. cholerae* [7]. Even in facultative intracellular pathogens such as *S. typhimurium* and *L. monocytogenes*, flagellar motility plays a role in optimal positioning on the host cell surface, thereby facilitating more efficient invasion [8,9].

Flagella are extracellular organelles that enable both swimming in liquid environments and swarming on surfaces [10,11]. Other surface-associated motility mechanisms include twitching via retractive type IV pili, as well as appendage-independent gliding and sliding [12]. Flagella are highly conserved among bacteria and are composed of distinct subcomplexes: an intracellular basal body (which includes the export apparatus and motor), and the extracellular hook and filament structures [12,13]. In Gram-negative bacteria, the export apparatus and rotor–stator complex are located in the cytoplasm, while the basal body forms a continuous structure spanning the periplasm and both the inner and outer membranes. Flagellar rotation is powered by a proton gradient across the membrane (the inner membrane in Gram-negative bacteria) [14].

The assembly of all intracellular and extracellular flagellar components depends on a highly conserved export apparatus, whose activity is essential for flagellum biogenesis. This export apparatus is evolutionarily related to the type III secretion system (T3SS) injectisome, which is characteristic of many Gram-negative pathogens [15,16]. Proteins fulfilling equivalent functions are highly similar between the flagellar export machinery and the T3SS injectisome [16].

Here, we investigated the effect of Fluorothiazinone (FT) on bacterial flagellar motility. FT is a small molecule of the 2,4-disubstituted-4H-[1,3,4]-thiadiazine-5-one class, previously shown to inhibit the T3SS [17,18,19]. FT has demonstrated therapeutic potential by suppressing infections caused by *P. aeruginosa*, *Salmonella enterica*, and UPEC [18,20,21]. Notably, not all of these pathogens utilize the T3SS during infection; for example, UPEC lacks a T3SS [21]. These findings suggest that FT’s in vivo antimicrobial effects may involve additional mechanisms beyond T3SS inhibition. Given the conserved nature of both the T3SS injectisome and the flagellar apparatus, we hypothesized that FT might also target flagellar motility. Indeed, FT has been shown to inhibit motility in UPEC [21]. In this study, we extended our investigation to assess the effects of FT on the motility of three Gram-negative and one Gram-positive bacterial species.

## 2. Results

### 2.1. Fluorothiazinone Inhibits Motility of Gram-Negative Bacteria

To study FT effects on bacterial motility, we tested three pathogenic species of Gram-negative bacteria: the *Pseudomonas aeruginosa* clinical strains GIMC5016:PA1840 and 2943; the *Proteus mirabilis* strains 1 and 172388, both isolated from patients with urological infections; and two *E. coli* strains: the uropathogenic *E. coli* (UPEC) strain GIMC1401:EC_CNL19 and enterohemorrhagic O157:H7 *E. coli* (EHEC) strain ATCC43890.

**Figure 1:**
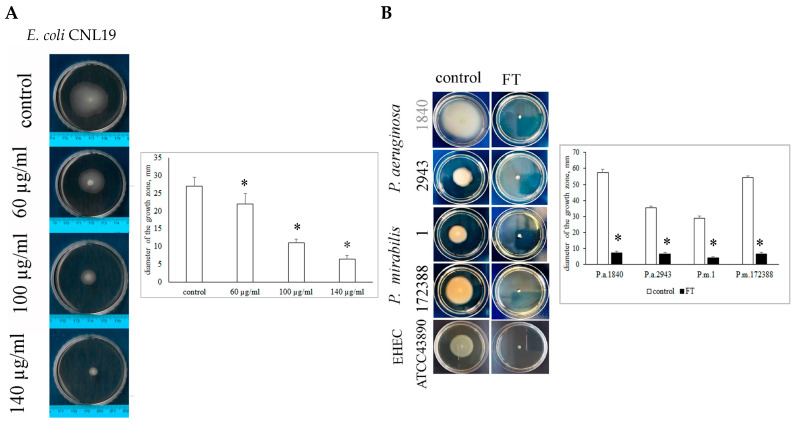
FT inhibits motility of Gram-negative bacteria in semisolid agar test. (**A**)—dose-dependent inhibition of the UPEC strain GIMC1401:EC_CNL19 motility in 0.25% agar; FT concentrations are shown; the spreading zone diameter is shown in mm. (**B**)—FT added to a semi-solid agar up to concentration of 100 µg mL^−1^ inhibits motility of *P. aeruginosa*, *P. mirabilis*, and UPEC6 strains; the spreading zone diameter is shown in mm. The median values and SD are displayed; *, *p* < 0.05.

First, we evaluated FT’s dose-dependent effects on the UPEC strain GIMC1401:EC_CNL19 using a semisolid agar motility test. Agar (0.25%) was supplemented with FT at concentrations of 30, 60, and 100 µg·mL^−1^ or left unsupplemented. The UPEC strain GIMC1401:EC_CNL19 culture, diluted up to 10^7^ colony-forming units (CFU)·mL^−1^, was inoculated into the center of a Petri dish. Bacterial motility was assessed by measuring the diameter of the spreading zone 24 h later. FT reduced the diameter by approximately 1.5-, 2.5-, and 4-fold at concentrations of 30, 60, and 100 µg·mL^−1^, respectively (Figure 1A). Thus, FT inhibited bacterial spreading through semisolid agar in a dose-dependent manner. Almost complete inhibition of motility was observed at 100 µg·mL^−1^ (Figure 1A).

To assess FT effects on other Gram-negative bacteria, *P. aeruginosa, P. mirabilis*, and EHEC were tested using the same agar assay. All bacteria were motile, demonstrating species-specific differences in the spreading zone diameter (Figure 1B, “control” lane). FT was added to a concentration of 100 µg·mL^−1^, and motility was assessed after 24 h. In all tested strains, 100 µg·mL^−1^ FT prevented bacterial spreading (Figure 1B, “FT” lane). Viability of bacteria incubated with FT for 24 h was confirmed by plating. These results demonstrate that FT inhibits motility in all tested Gram-negative bacteria.

### 2.2. FT Inhibits Bacterial Motility Using Microfluidic Chamber Technique

**Figure 2:**
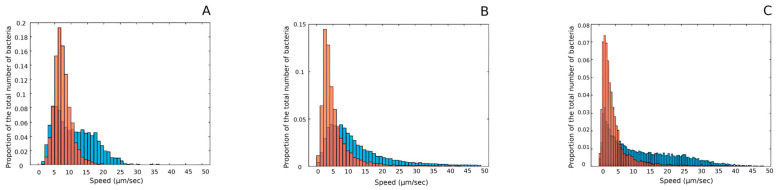
Distribution of individual velocities of *P. aeruginosa*. (**A**)—Individual velocities of the strain GIMC5016:PA1840 grown overnight in the LB broth supplemented with 100 µg mL^−1^ FT (pink) or in the unsupplemented LB broth (blue). (**B**)—Individual velocities of the strain 1840 grown overnight in the LB broth (blue) and bacteria from the same culture killed by heating (pink, Brownian motility). (**C**)—Individual velocities of the strain 1 grown overnight in the LB broth supplemented with 100 µg mL^−1^ FT (pink) or in the unsupplemented LB broth (blue).

To support the conclusion that FT inhibits bacterial motility and to obtain additional evidence of motility changes in its presence, we employed a recently developed in situ motility assay based on the analysis of bacterial cell motility within a microfluidic chamber [9,22]. To analyze FT effects on motility, the *P. aeruginosa* strain GIMC5016:PA1840 was grown overnight in LB broth supplemented with 100 µg·mL^−1^ FT or in unsupplemented LB broth. The overnight cultures were then adjusted to an optical density of OD_600_ ≈ 2.0, loaded into a microfluidic chamber with a channel height of 30 μm, and recorded at 400× magnification. Videos were analyzed to calculate individual velocities of 150–200 bacteria observed in the field of view over a 10 s interval. The distribution of individual velocities in FT-treated cells differed markedly from that of the untreated control (Figure 2A). Bacterial viability after incubation with FT was confirmed by plating. To determine whether the observed reduction in velocity indicated a complete loss of motility, we compared velocity distributions of viable and heat-killed *P. aeruginosa* cells (Figure 2B). Heat-killed bacteria exhibited Brownian motion with a distribution similar to that of FT-treated bacteria, supporting the conclusion that Fluorothiazinone effectively inhibits bacterial motility. A similar effect was observed in the *P. mirabilis* strain 1 following overnight FT treatment, indicating that FT impacts motility in a consistent manner across different Gram-negative species (Figure 2C).

### 2.3. L. monocytogenes Motility Is Not Affected by FT

**Figure 3:**
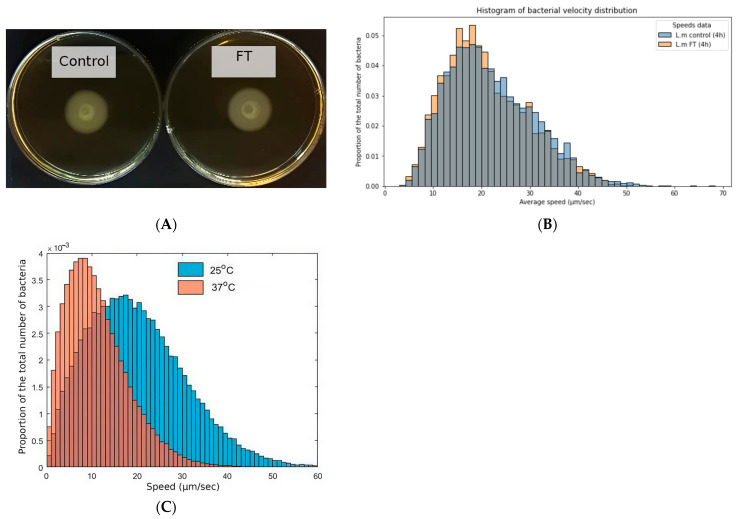
FT did not affect motility of *L. monocytogenes*. (**A**)—FT added to a semi-solid up to concentration of 100 µg mL^−1^ did not affect spreading of the *L. monocytogenes* strain EGDe. (**B**)—Distribution of individual velocities of *L. monocytogenes* grown at 25 °C in presence of FT (pink) and without FT (blue). (**C**)—Distribution of individual velocities of *L. monocytogenes* grown without FT at 25 °C (pink) and 37 °C (blue); *L. monocytogenes* grown at 37 °C lacks flagella and demonstrates Brownian motility [23].

To assess the breadth of bacteria susceptible to Fluorothiazinone’s effects on motility, we tested the Gram-positive bacterium *Listeria monocytogenes*. *L. monocytogenes* possesses peritrichous flagella, the expression of which is temperature-dependent. It is flagellated and motile when grown at room temperature but loses its flagella and becomes non-motile when grown at 37 °C [23]. We first tested the *L. monocytogenes* strain EGDe in a semisolid agar motility assay performed at 25 °C. There was no difference in the spreading of bacteria grown in the presence of 100 µg·mL^−1^ FT compared to the control (Figure 3A). Next, we compared the individual velocities of *L. monocytogenes* bacteria grown at 37 °C and at 25 °C, with and without Fluorothiazinone. The addition of FT did not alter the distribution of individual velocities in bacteria grown at 25 °C (Figure 3B), while it was distinct from the Brownian motion profile observed in bacteria grown at 37 °C (Figure 3C). These results suggest that Fluorothiazinone does not affect the motility of *L. monocytogenes*.

Comparing Brownian motility of *P. aeruginosa* grown in the presence of FT and *L. onocytogenes* grown at 37 °C demonstrated a clear difference in profiles of Brownian motility (compare Figure 2 and Figure 3C). This difference could be due to differences in bacterial sizes and eccentricity.

### 2.4. Consecutive Passages Do Not Promote Resistance to FT’s Effects on Motility

**Figure 4:**
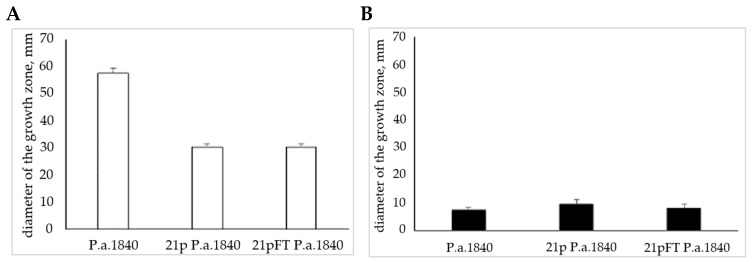
Repetitive re-plating of *P. aeruginosa* GIMC5016:PA1840 did not result in the appearance of resistance to FT. (**A**)—bacteria grown in a semi-solid agar; (**B**)—FT was added to semi-solid agar at concentrations up to 100 µg mL^−1^. P.a.1840—control bacteria from a fresh culture; 21p P.a.1840—bacteria from the 21st passage in LB without FT; 21pFT P.a.—bacteria from the 21st passage in LB supplemented with 100 μg mL^−1^ FT. The median values of the spreading zone diameter in mm and SD are shown.

To analyze whether resistance to FT could develop in terms of motility, the *P. aeruginosa* strain GIMC5016:PA1840 was consecutively passaged in LB broth supplemented with 100 μg·mL^−1^ FT. The control culture was passaged in LB without supplements. After 21 repetitive passages, bacterial motility was tested using a semisolid agar assay and in situ motility analysis. Repeated passaging caused a decrease in the diameter of the spreading zone in 0.25% agar (Figure 4). This decrease was similar for bacteria passaged with and without FT. The addition of FT to the soft agar inhibited motility of both control and passaged cultures in a similar manner, regardless of whether the cultures had previously been exposed to FT. For comparison, increased resistance to ampicillin appeared in the *E. coli* culture after 8–17 repetitive passages in the medium supplemented with a dose of ampicillin (50 μg/mL), which is comparable to therapeutic doses [24]. Resistance to sub-MIC streptomycin concentrations after 90 passages demonstrated 1% of bacteria of *S. typhimurium* culture, while the use of higher doses of some antibiotics leads to the appearance of resistant bacteria within a single passage [25].

### 2.5. Effect of Fluorothiazinone on Motility Requires at Least 2 h to Appear

**Figure 5:**
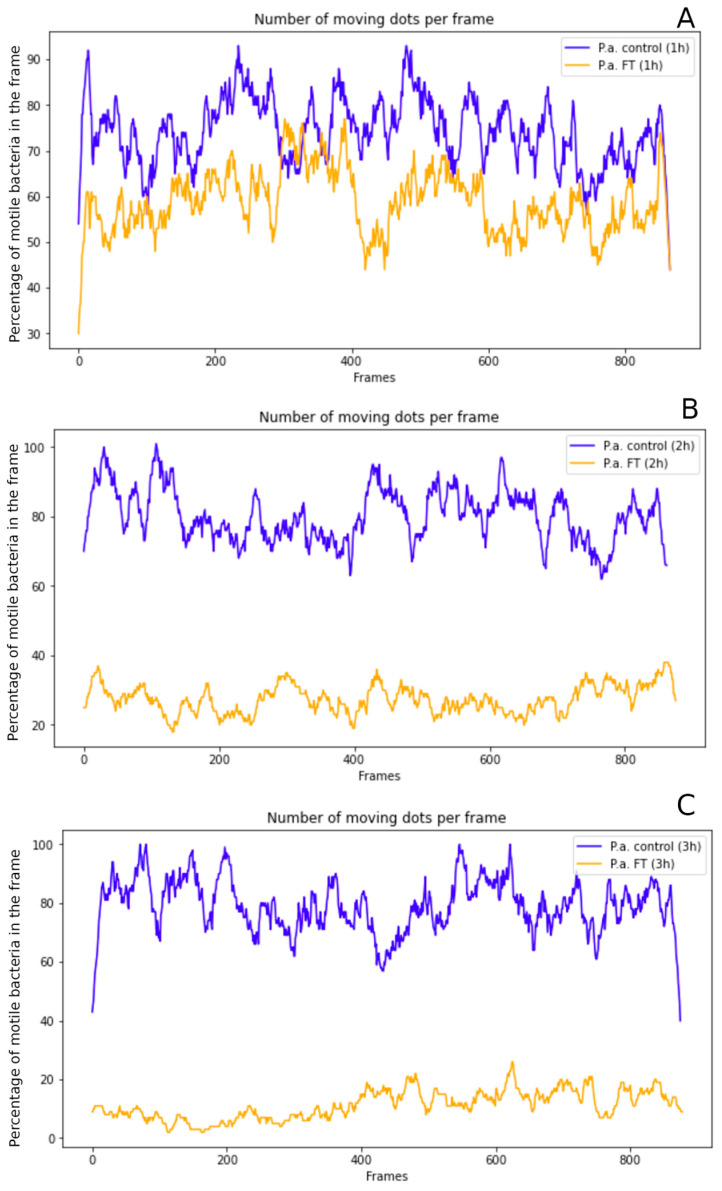
Time-dependence of FT effects on *P. aeruginosa* motility. The Y-axis shows the proportion of motile bacteria detected in each frame as a percentage (%). The total number of motile bacteria was estimated for the entire video. The overnight culture was diluted 1:100 and grown 2 h before FT was added up to 100 µg mL^−1^. The number of moving and motionless bacteria were calculated 1 h (**A**), 2 h (**B**), and 3 h (**C**) post FT addition.

The next question addressed was whether Fluorothiazinone inhibits flagellar rotation or affects flagellum synthesis and/or assembly. We determined the period required for motility loss after Fluorothiazinone addition. The *P. aeruginosa* strain GIMC5016:PA1840 grown overnight was diluted in fresh LB broth, and 100 µg·mL^−1^ FT was added 2 h after dilution. Motility was surveyed in situ every hour after dilution. Little change in the number of motile cells was observed within the first hour (Figure 5A). Two hours after FT addition, motionless bacteria prevailed in the culture supplemented with FT, while all bacteria in the control culture remained motile (Figure 5B). Similar results were observed three hours post FT addition (Figure 5C). These data suggested that FT does not have an immediate effect on bacterial motility. The lack of immediate effect may be due to the low rate of diffusion of FT into cells or the need for at least one division cycle for bacteria to lose motility after the addition of FT. The last explanation supports the hypothesis that FT inhibits flagellum assembly or synthesis rather than impairing its rotation.

### 2.6. Fluorothiazinone Inhibits Flagellum Assembly in Gram-Negative Bacteria

**Figure 6:**
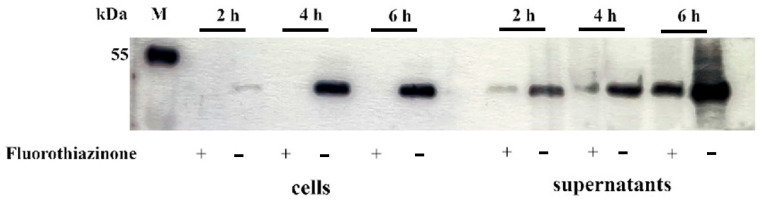
FT affects flagellin presentation on the surface of the EHEC O157:H7 strain ATCC43890. The overnight culture was diluted 1:100; FT was added to a final concentration of 100 µg·mL^−1^. Surface and secreted proteins were taken from a culture volume corresponding to 10^7^ CFU at 2, 4, and 6 h post-dilution, separated on 10% SDS-PAGE, transferred onto a nitrocellulose membrane, and tested with the anti-H7 antibody detected using an HRP-conjugated secondary antibody.

To gain further evidence on the mode of FT action, we analyzed the accumulation of the flagellar structural protein, flagellin, on the cell surface and in the supernatant during culture growth. The EHEC O157:H7 strain was used because of the availability of flagellin-specific antibodies. The overnight EHEC culture was diluted 1:100 in fresh LB broth with and without 100 µg·mL^−1^ FT and incubated at 37 °C with shaking. Using an H7 flagellin-specific antibody, cells and supernatants were checked for the presence of flagellin at 2, 4, and 6 h post-dilution. In control bacteria, cell-associated flagellin was detected starting at 2 h and later (Figure 6). The reduced level of cell-associated flagellin at the 2 h time point in the negative control seemed to be due to slow restoration of flagellin expression, which is repressed in a stationary culture *E. coli* [26]. FT completely inhibited the appearance of cell-associated flagellin at all time points. Meanwhile, free secreted flagellin was detected in the culture supernatant of both control and FT-supplemented cultures. The relative amount of secreted flagellin was noticeably higher in the control culture (Figure 6). These data suggested that FT inhibited flagellum assembly on the cell surface rather than its synthesis.

**Figure 7:**
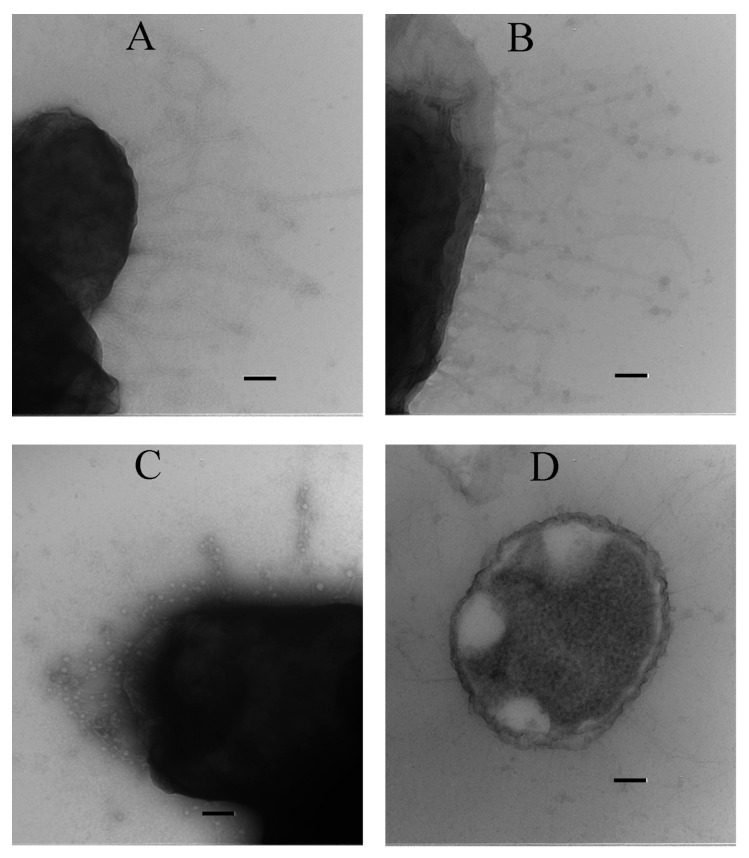
*P. mirabilis* grown in the presence of FT lacks flagella. *P. mirabilis* was grown overnight in LB (**A**,**B**) or in LB supplemented with 100 µg·mL^−1^ FT (**C**,**D**). Rare and short structures are seen on the surface of the bacteria grown in the presence of FT (**C**,**D**), while a dense network of long flagella can be seen in the control (**A**,**B**). Bar—100 nm.

To confirm this conclusion and to gather evidence that the mechanism of FT’s effect on motility is similar in other Gram-negative bacteria, we used electron microscopy to examine the presence of flagella on the surface of *P. mirabilis* grown overnight in LB broth supplemented or not with 100 µg·mL^−1^ FT. Flagella formed a noticeable network around control bacteria. The presence of FT significantly reduced the length and number of *P. mirabilis* flagella (Figure 7). These data support the hypothesis that FT prevents normal flagellum assembly on the bacterial surface.

## 3. Discussion

Recently, we have described Fluorothiazinone (FT), a low-molecular-weight molecule belonging to the 2,4-disubstituted-4H-[1,3,4]-thiadiazine-5-one class that targets T3SS and associated virulence without affecting bacterial viability [17,18,21]. FT was first demonstrated as a potent T3SS inhibitor in *Chlamydia trachomatis* and later confirmed in other models [17,18,20]. In mouse experiments, FT was shown to suppress infection by *P. aeruginosa*, *Salmonella enterica*, UPEC, *Acinetobacter baumannii*, and *Klebsiella pneumoniae* [18,20,27,28,29]. The clinical potential of FT was demonstrated in a clinical trial involving a total of 357 patients with complicated urinary tract infections (UTIs), which demonstrated its safety and efficacy when combined with cefepime compared to placebo/cefepime [19].

The wide activity of FT against Gram-negative bacteria was suggested to be due to the conserved nature of its molecular target, the T3SS ATPase involved in T3SS assembly and functioning [17]. However, in a mouse model, FT also prevents infection caused by bacteria that lack T3SS, including UPEC, *Acinetobacter baumannii*, and *Klebsiella pneumoniae* [21,30,31]. These data suggest that FT has additional targets beyond T3SS. Bacterial flagella may be one such target, as suggested by a previous study demonstrating an inhibitory FT effect on UPEC motility [21].

Here, we studied FT effects on bacterial motility in Gram-negative bacteria, including *E. coli*, *P. aeruginosa*, and *P. mirabilis*. To analyze bacterial motility, we used a semi-solid agar assay and in situ analysis in a microfluidic chamber. Both methods supported the conclusion about susceptibility of flagellar motility to FT. Thus, we demonstrated that FT inhibits motility of Gram-negative bacteria by disrupting flagellum assembly on the bacterial surface. Meanwhile, FT did not inhibit flagellar motility in the Gram-positive bacterium *L. monocytogenes*. Indeed, alignment of the proposed FT target, the conserved ATPase FliI, shows that the *L. monocytogenes* protein is divergent from its Gram-negative counterparts (Figure S1).

Flagellar motility plays an important role in bacterial virulence [4,32,33]. A number of efforts have been made to develop substances that inhibit flagellum rotation, regulation, and/or assembly. For instance, direct blocking of sodium-driven flagellar motors typical for some extremophiles was successful with pyrazine derivatives that inhibit the Na^+^ channels of the stator complexes, thereby preventing the generation of torque for flagellar rotation [34,35]. A benzoisothiazolinone derivative was found to block flagellum-dependent near-surface swarming by disrupting intracellular regulation of the second messenger cyclic diguanosine monophosphate (c-di-GMP) [36]. c-di-GMP, which represses bacterial flagella synthesis by interacting with the master regulator FleQ, is an important target for the development of novel classes of anti-infection substances [37,38].

The results obtained in this study demonstrate that FT affects neither rotation nor expression of flagellar components but rather the assembly of flagella on the bacterial surface. The following findings support this conclusion:

- The effect did not occur immediately after FT addition to motile bacteria but was delayed by at least 2–3 h—a time span too long for a direct effect on rotation but sufficient for bacterial division, resulting in progeny lacking flagella;
- Western blotting did not reveal cell-associated flagellin in bacteria grown in the presence of FT, although the structural filament protein was found in the supernatant. These data suggest that assembly, not expression, was affected. The decrease in secreted flagellin concentration might result from complex regulatory networks controlling early and late flagellar genes [39];
- Transmission electron microscopy confirmed that FT impaired flagellar presentation on the bacterial surface. The high homology between T3SS and the flagellar export machinery supports this hypothesis.

Taken together, our results demonstrate that Fluorothiazinone inhibits flagellum assembly in Gram-negative bacteria, thereby restricting bacterial motility. This mechanism may contribute to the inhibitory effects of Fluorothiazinone on bacterial infections in animal models and humans.

## 4. Materials and Methods

### 4.1. Bacterial Strains and Cultivation Conditions

Bacterial strains are listed in Table 1**.** Frozen bacterial stocks were plated onto agar and incubated at 37 °C. LB agar and broth (BD) were used to propagate Gram-negative bacteria, and Brain Heart Infusion (BHI, BD) was used to propagate *L. monocytogenes*.

### 4.2. Fluorothiazinone

Fluorothiazinone (FT), also known as CL-55, is N-(2,4-difluorophenyl)-4(3-ethoxy-4-hydroxybenzyl)-5-oxo-5,6-dihydro-4H-[1,3,4]-thiadiazine-2-carboxamide, synthesized as described previously [17].

### 4.3. Semisolid Agar Assay

Semisolid medium was prepared with 0.3% agar (Merck) and dispensed into Petri dishes. Bacterial cultures from fresh plates were inoculated in the center of the dish using a bacteriological loop. Opaque zones of bacterial spreading were observed 24 h post-inoculation. Zone diameters were measured. Experiments were performed in triplicate.

### 4.4. Development of FT Resistance Assay

To analyze whether resistance to FT could develop in terms of motility, the *P. aeruginosa* strain GIMC5016:PA1840 was consecutively passaged in LB broth supplemented with 100 μg·mL^−1^ FT. Briefly, overnight *P. aeruginosa* strain GIMC5016:PA1840 culture was diluted 1:100 in fresh LB with 100 μg·mL^−1^ FT to grow 24 h. Then the cycle was repeated with the obtained overnight culture. In parallel, the same initial culture was consecutively passaged in LB broth without FT. After 21 cycles, the bacteria froth overnight culture was plated on an LB plate, and the semisolid agar assay was performed as described above using LB agar plates with or without FT. As a control a freshly thawed but not passaged GIMC5016:PA1840 culture was used.

### 4.5. In Situ Motility Assay

The observation and characterization of bacterial motility were performed as described previously [22]. The overnight cultures were diluted 1:19 in fresh broth and grown; to the late exponential phase. Optical density was adjusted to OD_600_ = 2.0, corresponding to a cell density of ≈1 × 10^9^ CFU mL^−1^. FT was added immediately to the diluted culture if another is not stated.

The diluted culture was placed in a PLA microfluidic chamber with a 30 μm deep × 10 mm wide channel. A glass coverslip was pressed onto the chamber surface. A series of 30 s videos of moving bacteria were recorded at 30 frames per second by brightfield microscopy using a Zeiss Axio Scope A1 microscope (Carl Zeiss AG, Oberkochen, Germany), equipped with the Achroplan 40×/0.65 objective and DCM510 CMOS digital camera (5 MPx, Hangzhou, China), with resolution up to 2560 × 1920 px. At least 5 videos were recorded per sample. All experiments were repeated at least 3 times. Observations were made at approximately 15 μm above the channel bottom.

Approximately 10 s video segments were selected for analysis. Videos were split into frames and converted to grayscale. Background subtraction was performed using a sliding average method, with each frame’s background calculated based on neighboring frames. Bright features corresponding to bacteria were identified; only features exceeding a set intensity threshold were analyzed.

Detected features were linked across frames using a proximity-based algorithm that assigned consistent trajectory IDs based on a maximum displacement parameter. Instantaneous velocities were calculated, and mean velocities were derived. Velocity histograms were constructed.

The algorithm was implemented in Python 3.11 using open-source libraries, including trackpy, pandas, matplotlib, seaborn, numpy, and pims. Detection and tracking were validated by visual inspection of selected frames and analysis of trajectory properties such as duration and particle count with various parameter settings.

### 4.6. Isolation of Surface-Associated and Secreted E. coli Proteins

The overnight *E. coli* ATCC 43890 culture was diluted 1:19 in LB broth and grown at 37 °C with shaking at 200 rpm. Samples containing approximately 2.5 × 10^10^ CFU were taken at 2, 4, and 6 h post-dilution. Bacteria were centrifuged at 4200 rpm, and cells and supernatants were processed separately.

To obtain secreted proteins, trichloroacetic acid (TCA) was added to the supernatant to a final concentration of 8%, and the mixture was incubated on ice for 1 h. Proteins were pelleted by centrifugation at 6000 rpm for 10 min and washed 3 times with ice-cold 70% ethanol. The precipitate was then dissolved in 1× Laemmli buffer (0.0625 M Tris-HCl, pH 6.8, 2% SDS, 10% glycerol, 5% β-mercaptoethanol, and 0.002% bromophenol blue) and boiled for 5 min.

Surface-attached flagellin was isolated as described in [42]. Briefly, precipitated cells were resuspended in 300 μL of A-solution (150 mM NaCl, 10 mM HCl) and incubated at room temperature with shaking at 150 rpm for 30 min. The pH was adjusted to 7.0 with 50 mM Tris and 10 mM NaOH. The suspension was clarified by centrifugation at 11,000 rpm for 10 min. The supernatant was mixed with 4× Laemmli buffer (1:3) and boiled for 5 min before SDS-PAGE.

### 4.7. SDS-PAGE and Western Blotting

Secreted and surface-associated proteins were separated on 10% SDS-PAGE and transferred to a PVDF membrane (Elabscience Houston, TX, USA). The membrane was blocked with 2% BSA for 1 h at room temperature then incubated with a recombinant human monoclonal anti-*E. coli* O157:H7 FliC/flagellin antibody (Cell Sciences, Newburyport, MA, USA) diluted 1:5000 for 1 h. The membrane was washed with TTBS (20 mM Tris-HCl, pH 7.5, 150 mM NaCl, 0.1% Tween-20).

A secondary anti-human HRP-conjugated antibody (Thermo Fisher Scientific, Waltham, MA, USA) was added at 1:50,000 dilution, incubated for 1 h, and the membrane was washed with TTBS. Flagellin was visualized using the SuperSignal™ West Dura Extended Duration Substrate (Thermo Fisher Scientific) and Hyperfilm™ ECL (Amersham Biosciences Corp., Woburn, MA, USA).

### 4.8. Transmission Electron Microscopy with Negative Staining

A drop of sample suspension was placed on a formvar-coated and carbon-evaporated 200 mesh copper grid (EMS, Hatfield, PA, USA) for 1 min. Excess fluid was removed with filter paper. A drop of 2% uranyl acetate in 0.2 M maleate buffer was applied for 1 min then removed. Samples were dried for 10 min at room temperature and analyzed using a JEM 2100Plus transmission electron microscope (JEOL Ltd, Tokyo, Japan) at 160 kV.

## 5. Conclusions

Here, we demonstrated that a small molecule of the 2,4-disubstituted-4H-[1,3,4]-thiadiazine-5-one class, Fluorothiazinone (FT), inhibits the flagellar motility of Gram-negative pathogenic bacteria, including *E. coli*, *P. aeruginosa*, and *P. mirabilis*, but not of Gram-positive *L. monocytogenes*. Taken together, the obtained results suggested that FT inhibits an assembly of flagella on the bacterial surface rather than flagellar rotation. Motility inhibition in pathogenic bacteria can contribute to FT therapeutic effects on Gram-negative bacterial infections in both humans and animals.

## Figures and Tables

**Table 1:** Bacterial strains used in the study.

Strain	Characteristics	Reference
*Escherichia coli*		
GIMC1401:EC_CNL19	UPEC, clinical isolate	[21]
ATCC 43890	EHEC, O157:H7, type strain	[40]
*Pseudomonas aeruginosa*		
GIMC5016:PA1840	Clinical isolate	
2943	Clinical isolate	
*Proteus mirabilis*		
1	Clinical isolate	
172388	Clinical isolate	
*Listeria monocytogenes*		
EGDe	Type strain	[41]

## Data Availability

The original contributions presented in this study are included in the article/Supplementary Material. Further inquiries can be directed to the corresponding author.

## Supplementary Materials

The following supporting information can be downloaded at: https://www.mdpi.com/article/10.3390/antibiotics14080820/s1. Figure S1: Alignment of flagellar ATPase FilI. Original data (Bacterial_motility) can be downloaded at https://github.com/alexslonik/Bacterial_motility (accessed on 30 May 2025).

## Abbreviations

The following abbreviations are used in this manuscript:

FT	Fluorothiazinone—a small molecule of the 2,4-disubstituted-4H-[1,3,4]-thiadiazine-5-one class
LB	Luria-Bertani Broth
UPEC	Uropathogenic *Escherichia coli*
EHEC	Enterohemorrhagic *Escherichia coli*
