# Supplementary material for: The Small Molecule Inhibitor of the Type III Secretion System Fluorothiazinone Affects Flagellum Surface Presentation and Restricts Motility in Gram-Negative Bacteria
Source: Antibiotics (Basel). 2025 Aug 11;14(8):820. doi: 10.3390/antibiotics14080820 (PMC12382710; doi:10.3390/antibiotics14080820)
Supplement: Supplementary file 1 [file antibiotics-14-00820-s001.zip › antibiotics-3774439-supplementary.pdf]

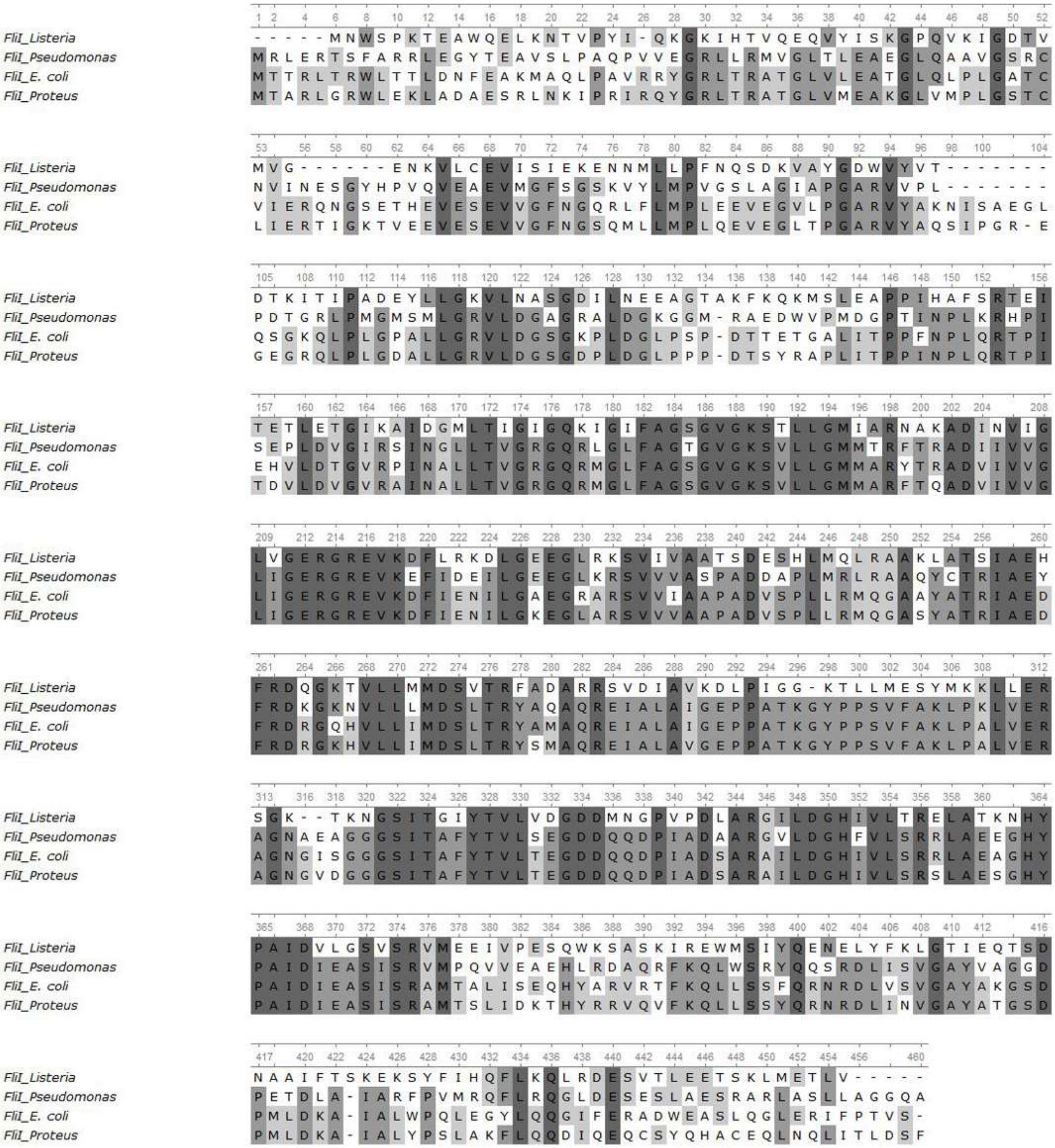

Fig. S1. Alighment of falgellar ATPase FliI. The foloowing sequences were used^
KXC00516.1 flagellar protein export ATPase FliI [Proteus mirabilis]
BFN10064.1 flagellum-specific ATP synthase FliI [Pseudomonas aeruginosa]
ACA77353.1 ATPase, FliI/YscN family [Escherichia coli ATCC 8739]
WP_003724451.1 flagellar protein export ATPase FliI [Listeria monocytogenes]
